# Rheumatologists’ adherence to a disease activity score steered treatment protocol in early arthritis patients is less if the target is remission

**DOI:** 10.1007/s10067-016-3405-8

**Published:** 2016-09-28

**Authors:** G. Akdemir, I.M. Markusse, Y.P.M. Goekoop-Ruiterman, G.M. Steup-Beekman, B.A.M. Grillet, P.J.S.M. Kerstens, W.F. Lems, T.W.J. Huizinga, C.F. Allaart

**Affiliations:** 1 Department of Rheumatology, Leiden University Medical Center, PO BOX 9600, 2300 RC Leiden, The Netherlands; 2 Haga Hospital, The Hague, Netherlands; 3 Bronovo Hospital, The Hague, Netherlands; 4 Zorgsaam, Terneuzen, Netherlands; 5 Reade, Amsterdam, the Netherlands; 6 VUMC, Amsterdam, the Netherlands

**Keywords:** DAS steered protocols, Early rheumatoid arthritis, Physician, Protocol adherence, Treatment target

## Abstract

To compare rheumatologists’ adherence to treatment protocols for rheumatoid arthritis (RA) targeted at Disease Activity Score (DAS) ≤2.4 or <1.6. The BeSt-study enrolled 508 early RA (1987) patients targeted at DAS ≤2.4. The IMPROVED-study included 479 early RA (2010) and 122 undifferentiated arthritis patients targeted at DAS <1.6. We evaluated rheumatologists’ adherence to the protocols and assessed associated opinions and conditions during 5 years. Protocol adherence was higher in BeSt than in IMPROVED (86 and 70 %), with a greater decrease in IMPROVED (from 100 to 48 %) than in BeSt (100 to 72 %). In BeSt, 50 % of non-adherence was against treatment intensification/restart, compared to 63 % in IMPROVED and 50 vs. 37 % were against tapering/discontinuation. In both studies, non-adherence was associated with physicians’ disagreement with DAS or with next treatment step and if patient’s visual analogue scale (VAS) for general health was ≥20 mm higher than the physician’s VAS. In IMPROVED, also discrepancies between swelling, pain, erythrocyte sedimentation rate, and VASgh were associated with non-adherence. Adherence to DAS steered treatment protocols was high but decreased over 5 years, more in a DAS <1.6 steered protocol. Non-adherence was more likely if physicians disagreed with DAS or next treatment step. In the DAS <1.6 steered protocol, non-adherence was also associated with discrepancies between subjective and (semi)objective disease outcomes, and often against required treatment intensification. These results may indicate that adherence to DAS-steered protocols appears to depend in part on the height of the target and on how physicians perceive the DAS reflects RA activity.

**Electronic supplementary material:**

The online version of this article (doi:10.1007/s10067-016-3405-8) contains supplementary material, which is available to authorized users.

## Introduction

The optimal treatment strategy to suppress disease activity in early arthritis patients is by initial combination therapy followed by targeted treatment [1–7]. Although in clinical trials, treat-to-target therapy has been already widely used, implementation in daily practice appears to be difficult [8–10]. Furthermore, it is unknown what the optimum treatment target is. It is recommended to aim at disease activity score (DAS)-remission (<1.6) or low disease activity (DAS <2.4). [11] A lower disease activity seems to be the optimal treatment target with better disease outcomes. [4, 7] However, achieving lower DAS and having better disease outcomes may not be causally related but results of mutually interdependent qualities or characteristics. Remission, especially by the strictest definition, can be difficult to achieve in daily practice. Moreover, steering at remission when disease activity is already low can lead to more costs and side effects with no added clinical benefit. Rheumatologists may be reluctant to aim for remission if disease activity is already substantially decreased from baseline, especially if they feel that the measured DAS is falsely elevated due to symptoms or inflammation not caused by rheumatoid disease activity.

We tried to estimate rheumatologists’ willingness and arguments to treat-to-target if the target was low disease activity or DAS-remission by comparing two clinical trials in patients with RA where the treatment targets were DAS ≤2.4 and DAS <1.6. The BeSt-study, a multicenter randomized clinical trial set up in the year 2000, when treat-to-target was not yet part of daily practice. Four different treatment strategies were assessed in early rheumatoid arthritis (RA) patients aiming at low disease activity (DAS ≤2.4). Seven years later in rheumatology centers who also participated in the BeSt-study, the IMPROVED-study started, a randomized clinical trial. Early RA and undifferentiated arthritis (UA) patients were treated with methotrexate (MTX) and tapered high dose of prednisone followed by treatment targeted at DAS-remission (DAS <1.6). To investigate whether these treatment targets can be equally well implemented in daily practice, we compared rheumatologists’ adherence to these DAS-steered treatment protocols targeted at either DAS ≤2.4 or DAS <1.6 and assessed associated opinions of the rheumatologists and conditions that may result in non-adherence by the rheumatologist during 5 years follow-up.

## Materials and methods

### Study design and patients

The BeSt-study (Dutch acronym for treatment strategies) was a multicenter, randomized, clinical trial started in 20 hospitals in the Netherlands in the year 2000, when treat-to-target was not daily practice. The aim was to evaluate the efficacy of four treatment strategies in 508 early active RA according to the 1987 American College of Rheumatology (ACR) criteria [12]. Every 3 months, the DAS was measured and calculated by the research nurse, and treatment adjustments were initiated by the rheumatologist targeted at low disease activity (DAS ≤2.4). If patients did not achieve low disease activity, the next treatment step was taken (supplementary Fig. 1). If the DAS was ≤2.4 for at least 6 months, medication was tapered to a maintenance dose. From year 2, if next the DAS was <1.6 for at least 6 months, medication was discontinued, but when the DAS was ≥1.6 medication was restarted, and subsequently increased or tapered depending on the DAS as mentioned above. The study was approved by the Medical Ethics Committee of each participating center and all patients gave written informed consent. More details about the BeSt-study were previously published [3, 5].

The IMPROVED-study (acronym for Induction therapy with MTX and prednisone in rheumatoid or very early arthritic disease) was a multicenter, randomized, clinical trial started in 2007 in 12 hospitals in the western part of the Netherlands, who also participated in the BeSt-study. 479 early RA according to the 2010 ACR and European League Against Rheumatism (EULAR) classification criteria [13] and 122 UA patients, started with induction therapy with MTX and tapered high dose of prednisone followed by 4-monthly treatment targeted at DAS-remission (<1.6). If patients were in DAS-remission, the medication was tapered and finally stopped but if DAS was >1.6, the medication was intensified or restarted (supplementary Fig. 2). All patients gave written informed consent and the Medical Ethical Committee of each participating center approved the study protocol. Details about the IMPROVED-study were published elsewhere. [4, 7].

### Measurements

All treatment steps in both studies were recorded in two different databases. We evaluated whether each treatment step was by protocol or not. Every study visit, the rheumatologist was asked to fill out a brief questionnaire about satisfaction with the effect of treatment, agreement with the required treatment step, and agreement with the DAS (Table 1). Also, the rheumatologists recorded their estimation of the patient’s disease activity on a visual analogue scale (VASphys, 0–100 mm, 0= inactive, 100= most active).

**Table 1:**
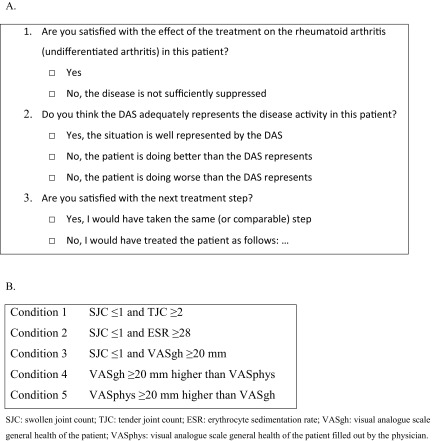
A. brief questionnaire filled out by the physician at every visit, B. five hypothetical conditions. *SJC* swollen joint count; *TJC* tender joint count; *ESR* erythrocyte sedimentation rate; *VASgh* visual analogue scale general health of the patient; *VASphys* visual analogue scale general health of the patient filled out by the physician

Five hypothetical conditions were formulated that may have an effect in the decision process of the rheumatologist to take a treatment step not by protocol [14]. These conditions aim to represent likely discrepancies between synovitis observed at physical examination and reported pain at physical examination or signs of inflammation in the laboratory analysis and discrepancies between the VASphys and the VAS for global health by the patient (VASgh) as used in calculation of the DAS (Table 1).

### Statistical analyses

Data of 5 years follow-up from both studies were used. Both studies were compared for frequency of adherence and protocol violations using descriptive statistics. A generalized linear mixed model (GLMM) for each study was used to evaluate: the association between protocol violations and the answers to the rheumatologists’ questionnaire; the association between protocol violations (dependent) and the presence of the hypothetical conditions (independent); the association between the (dis)agreement with the DAS as filled out in the questionnaire by the rheumatologist (dependent) and the presence of the hypothetical conditions (independent); the association between the (dis)agreement with the DAS as filled out on the questionnaire by the rheumatologist (dependent) and DAS categories (independent) (For the BeSt-study, three DAS categories were used (DAS-remission <1.6, low disease activity ≥1.6 ≤ 2.4, and high disease activity >2.4) and for the IMPROVED-study, two categories were used (DAS-remission <1.6, and no DAS-remission ≥1.6)); the association between physician’s satisfaction with how effect of treatment (dependent) and DAS categories as mentioned above (independent). An autoregressive moving average was used for the correlation matrix in both studies that assumes that observations that are further apart are less strongly correlated. Statistical analyses were performed with SPSS for Windows version 23.0.

## Results

### Protocol adherence and violations

**Fig. 1:**
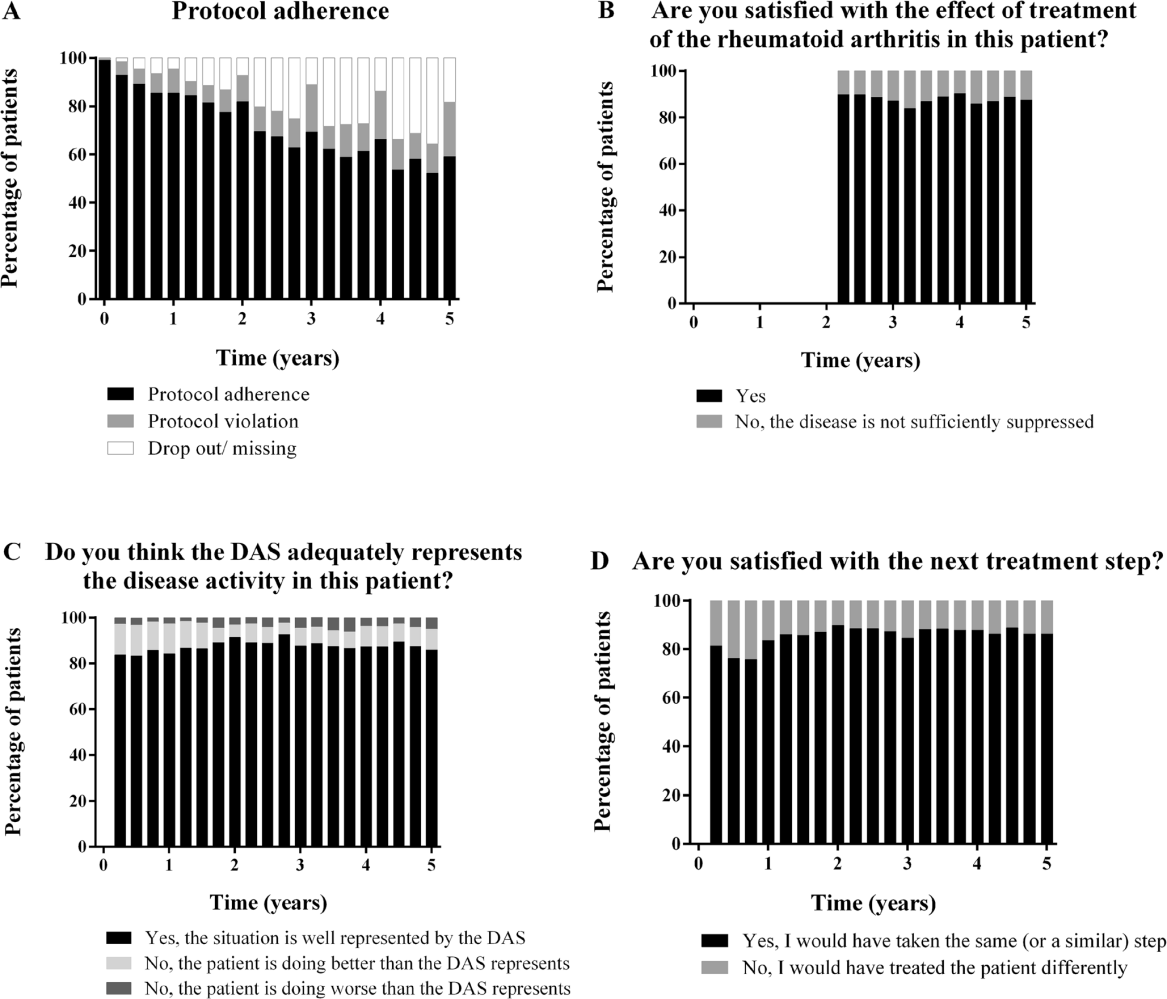
Protocol adherence and violations in the BeSt-study and answers of the rheumatologist to the questionnaire**. a**: protocol adherence was evaluated every visit; **b**: question was asked every visit from the tenth visit in year 3 until the end of follow-up; **c**: question was asked every visit from the second visit until the end of follow-up; **d**: question was asked every visit from the second visit until the end of follow-up. *DAS* disease activity score

**Fig. 2:**
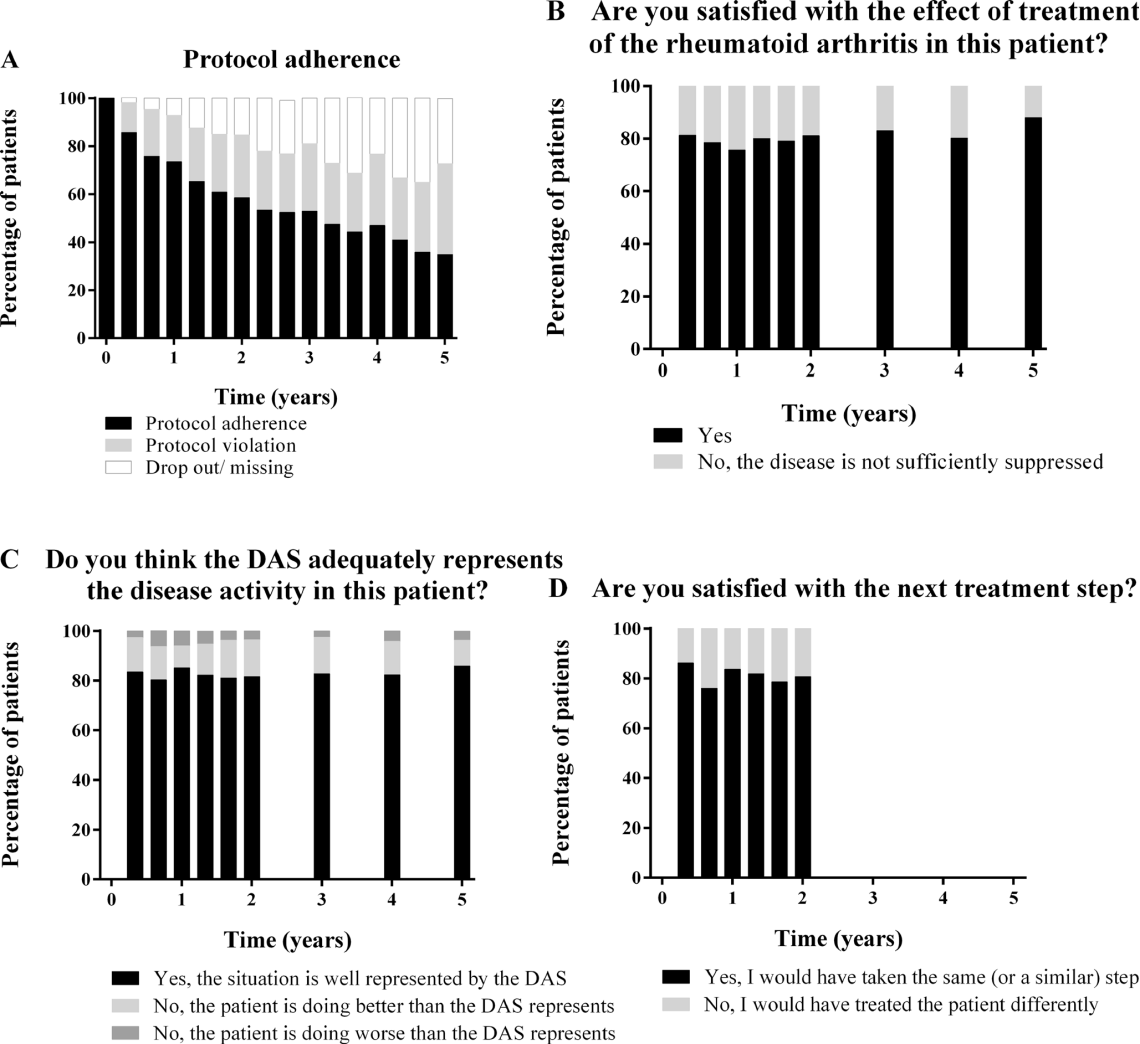
Protocol adherence and violations in the IMPROVED-study and answers of the rheumatologist to the questionnaire**. a**: protocol adherence was evaluated every visit; **b**: question was asked every visit from the second visit and after the second year only at yearly visits; **c**: question was asked every visit from the second visit and after the second year only at yearly visits; **d**: question was asked every visit from the second visit until the seventh visit. *DAS* disease activity score

Frequencies of protocol adherence and violations per visit during 5 years follow-up are shown in Fig. 1a for the BeSt-study and in Fig. 2a for the IMPROVED-study. Of the visit at *t* = 5 years, data were available for 82 % of patients in the BeSt-study and in 73 % in the IMPROVED-study. Rheumatologists’ adherence to the protocol was greater in the BeSt-study than in the IMPROVED-study in completed visits up to the fifth year (mean over time 86 and 70 %, respectively). Protocol adherence decreased over time from 100 to 72 % in the BeSt-study and from 100 to 48 % in the IMPROVED-study. Protocol violations could entail either omitting to restart or intensify medication (as required if DAS was above treatment target: high DAS protocol violation) or omitting to taper or stop (as required if DAS was below treatment target: low DAS protocol violation). Of all protocol violations in the BeSt-study, 50 % were low-DAS protocol violations and 50 % were high-DAS protocol violations. In case of a high-DAS protocol violation, the measured DAS was (median) 0.6 (interquartile range IQR 0.3;1.2) higher than the target DAS, whereas the difference was 0.9 (0.4;1.6) when the protocol for high DAS was followed (Table 2). In case of a low-DAS protocol violation, the measured DAS was 0.7 (−1.2;-0.3) below the target DAS, whereas the difference was −0.9 (−1.4;-0.5) when the protocol for low DAS was followed. Patients’ age was associated with more high-DAS protocol violations (1.02 (1.01–1.03)), and gender showed a trend (female gender 1.44 (0.94–2.21)), but these associations were not found for low-DAS protocol violations. There was no difference in protocol violations between the treatment arms (*p* = 0.872). In both studies, physicians in the peripheral centers had higher adherence compared to those in the two university centers (BeSt-study 95 % peripheral vs 87 % university and IMPROVED-study 94 vs. 66 %, respectively).

**Table 2:** Differences with DAS-target in protocol violations (high-DAS or low-DAS) and no protocol violations

	BeSt-study target 2.4				IMPROVED-study Target 1.6			
	High DAS PV	No PV	Low DAS PV	No PV	High DAS PV	No PV	Low DAS PV	No PV
mDAS, mean ± SD	3.2 ± 0.7	3.5 ± 0.9	1.6 ± 0.6	1.5 ± 0.6	2.2 ± 0.5	2.4 ± 0.6	1.0 ± 0.4	0.9 ± 0.4
Delta mDAS tDAS, mean ± SD median (IQR)	0.8 ± 0.7 0.6 (0.3;1.2)	1.1 ± 0.9 0.9 (0.4;1.6)	−0.8 ± 0.6 −0.7 (−1.2;−0.3)	−0.9 ± 0.6 −0.9 (−1.4;−0.5)	0.6 ± 0.5 0.5 (0.2;0.9)	0.8 ± 0.6 0.7 (0.3;1.2)	−0.6 ± 0.4 −0.6 (−0.9;−0.3)	−0.7 ± 0.4 −0.7 (−1.0;−0.4)

*DAS* disease activity score, *PV* protocol violation, *mDAS* measured DAS, *tDAS* target DAS

Of all protocol violations in the IMPROVED-study, 63 % were high-DAS protocol violations and 37 % were low-DAS protocol violations. In case of a high-DAS protocol violation, the measured DAS was (median) 0.5 (IQR 0.2;0.9) higher than the target DAS, whereas the difference was 0.7 (0.3;1.2) when the protocol for high DAS was followed. In case of a low-DAS protocol violation, the measured DAS was −0.6 (−0.9;−0.3) lower than the target DAS, whereas the difference was −0.7 (−1.0;−0.4) when the protocol for low DAS was followed. Patient’s gender was associated with high-DAS protocol violations (OR for females 1.53 (1.23–1.90)) and age showed a trend (1.01 (1.00–1.02)). Age and gender were not associated with low-DAS protocol violations. Diagnosis of RA (OR 1.47 (1.05–2.06)) and treatment group (arm 1 OR 2.07 (1.36–3.13) and arm 2 OR 1.87 (1.21–2.87)) were associated with more low DAS-protocol violations, and diagnosis RA was associated with *fewer* high-DAS protocol violations (OR 0.73 (0.56–0.95)). Both arm 1 (OR 1.44 (1.13–1.85)) and arm 2 (1.69 (1.31–2.19)) were also associated with more high-DAS protocol violations in the IMPROVED-study. As expected, there were more protocol violations in the outside of protocol group (OR for high-DAS protocol violations 2.84 (2.05–3.94)).

In the BeSt-study, rheumatologists were more likely not to follow the protocol if they were not satisfied with the current treatment effect (OR (95 % CI) 1.36 (1.08–1.71)), disagreed with how the DAS represented actual disease activity (2.26 (1.84–2.78) when they thought the DAS overestimated disease activity and 2.82 (2.08–3.81) when they thought the DAS underestimated disease activity), were not satisfied with the current treatment effect (OR (95 % CI) 1.36 (1.08–1.71)) or disagreed with the next treatment step (2.77 (2.34–3.28)) (Table 3). However, in 346/463 (75 %) visits where the rheumatologist was not satisfied with the current treatment effect the protocol was still followed, as also occurred in 714/939 (76 %) visits where the rheumatologist disagreed with how the DAS represented actual disease activity, and in 832/1070 (78 %) visits where the rheumatologist did not agree with the next treatment step.

**Table 3:** GLMM outcomes with protocol violation as dependent variable and opinions and conditions as independent variables

BeSt				IMPROVED		
Opinions	OR	95 % CI	*p* value	OR	95 % CI	*p* value
Not satisfied with treatment effect	1.36	1.08–1.71	0.010	0.59	0.49–0.72	<0.001
Disagreement with DAS (felt overestimation of actual disease activity)	2.26	1.84–2.78	<0.001	5.97	4.82–7.40	<0.001
Disagreement with DAS (felt underestimation of actual disease activity)	2.82	2.08–3.81	<0.001	1.44	1.01–2.07	0.047
Not satisfied next treatment step	2.77	2.34–3.28	<0.001	3.53	2.84–4.37	<0.001
Conditions						
1	1.00	0.85–1.18	0.993	3.1	2.73–3.52	<0.001
2	1.03	0.80–1.33	0.826	1.74	1.42–2.14	<0.001
3	1.04	0.89–1.22	0.629	2.03	1.80–2.29	<0.001
4	1.34	1.14–1.57	<0.001	2.18	1.85–2.56	<0.001
5	1.36	1.00–1.86	0.050	0.89	0.64–1.24	0.493

*DAS* disease activity score, *OR* odds ratio, *CI* confidence interval

Compared to the BeSt-study, in the IMPROVED-study a protocol violation appeared even more likely if rheumatologists disagreed with how the DAS represented actual disease activity (5.97 (4.82–7.40) if they thought the DAS overestimated disease activity and 1.44 (1.01–2.07) if they thought the DAS underestimated disease activity) or disagreed with the next treatment step (3.53 (2.84–4.37)). However, if they were not satisfied with the current treatment effect, this was associated with fewer protocol violations (0.59 (0.49–0.72)). In 299/647 (46 %) visits, there was still protocol adherence although the rheumatologist disagreed with the DAS, as in 280/475 (59 %) visits where the rheumatologist was not satisfied about the next treatment step and 565/736 (77 %) visits where the rheumatologists were not satisfied with the effect of current treatment.

When testing the five hypothetical conditions, in the BeSt-study more protocol violations were likely if the VASgh was ≥20 mm higher than the VASphys (1.34 (1.14–1.57)) (condition 4, Table 1). In the IMPROVED-study, this association was also found (2.18 (1.85–2.56)). In addition, the risk of a protocol violation was also higher if the swollen joint count (SJC) was ≤1 but tender joint count (TJC) was ≥2 (3.1 (2.73–3.52)) (condition 1, Table 1) or SJC was ≤1 and the erythrocyte sedimentation rate (ESR) was ≥28 (1.74 (1.42–2.14)) (condition 2, Table 1), and or SJC was ≤1 and VAS patient was ≥20 (2.03 (1.80–2.29)) (condition 3, Table 1). In the BeSt-study, these associations were not found.

### Agreement with how the DAS represents actual disease activity in relation to treatment targets

The rheumatologists answered that the actual disease activity was well represented by the DAS in 87 % of visits in the BeSt-study and 83 % in the IMPROVED-study (Figs. 1c and 2c). If misrepresentation of actual disease activity was suspected, the rheumatologists mostly felt that the patient was doing better than the DAS indicated and only rarely did they report to feel that the measured DAS underestimated actual disease activity. In the BeSt-study, the higher the DAS, the more likely that rheumatologists suspected overestimation of disease activity (by category: DAS >2.4: 97.29 (58.45–161.93), DAS ≥1.6 but ≤2.4: 9.86 (5.88–16.53)) (Table 4). Also as a continuous variable, a higher DAS was associated with more reports of DAS overestimating actual disease activity (2.97 (2.72–3.24)). In the IMPROVED-study, a DAS ≥1.6 was more often associated with reports of overestimated actual disease activity (22.03 (16.65–29.15), for DAS as a continuous variable 3.68 (3.25–4.16)).

**Table 4:** GLMM outcomes with DAS over/underestimation as dependent variable and DAS and conditions as independent variables

BeSt				IMPROVED		
Dependent: DAS overestimation	OR	95 % CI	*p* value	OR	95 % CI	*p* value
DAS <1.6	ref			ref		
DAS ≥1.6–≤ 2.4	9.86	5.88–16.53	<0.001	22.03	16.65–29.15	<0.001
DAS >2.4	97.29	58.45–161.93	<0.001			
DAS	2.97	2.72–3.24	<0.001	3.68	3.25–4.16	<0.001
Conditions						
1	0.87	0.73–1.03	0.096	5.65	4.67–6.84	<0.001
2	1.16	0.88–1.52	0.300	1.88	1.39–2.55	<0.001
3	0.76	0.64–0.91	0.002	3.03	2.51–3.66	<0.001
4	2.96	2.51–3.49	<0.001	4.49	3.68–5.48	<0.001
Dependent: DAS underestimation						
DAS <1.6	0.53	0.40–0.70	<0.001	0.48	0.38–0.60	<0.001
DAS ≥1.6–≤ 2.4	1.40	1.11–1.77	0.005	ref		
DAS >2.4	ref					
DAS	1.39	1.25–1.55	<0.001	1.95	1.70–2.25	<0.001
Condition						
5	6.73	5.00–9.06	<0.001	8.21	5.80–11.61	<0.001
Dependent: satisfied with treatment effect						
DAS <1.6	76.48	53.67–108.98	<0.001	26.06	20.68–32.84	<0.001
DAS ≥1.6–≤ 2.4	10.07	7.95–12.76	<0.001	ref		
DAS >2.4	ref					
DAS	0.09	0.08–0.11	<0.001	0.07	0.06–0.08	<0.001

*DAS* disease activity score, *OR* odds ratio, *CI* confidence interval

Both in the BeSt-study and the IMPROVED-study, rheumatologists were more likely to report that the DAS overestimated actual disease activity if VASgh was ≥20 mm higher than the VASphys (condition 4) (Table 4). If SJC ≤1 and VASgh ≥20 mm (condition 3) (0.76 (0.64–0.91)) in the BeSt-study DAS overestimation was less often reported, in contrast to the IMPROVED-study where this condition was associated with more DAS overestimation (3.03 (2.51–3.66)). In the IMPROVED-study, the rheumatologists answered that there was a DAS overestimation if SJC ≤1 and TJC ≥2 (condition 1) (5.65 (4.67–6.84)) and SJC ≤1 and ESR ≥28 (condition 2) (1.88 (1.39–2.55)).

DAS underestimation was filled out by the rheumatologists if the DAS was higher in the BeSt-study (category ≥1.6–≤2.4 (1.40 (1.11–1.77)), category DAS <1.6 (0.53 (0.40–0.70))) (Table 4). In the IMPROVED-study if the DAS was <1.6, the rheumatologists did not feel that the DAS was underestimating the disease activity (0.48 (0.38–0.60)). Increase in DAS was associated with more DAS underestimation in both studies (BeSt-study: 1.39 (1.25–1.55) and IMPROVED-study 1.95 (1.70–2.25)). Condition 5 (VASphys ≥20 mm higher than VASgh) was in both studies associated with DAS underestimation (6.73 (5.00–9.06) BeSt-study and 8.21 (5.80–11.61) IMPROVED-study).

### Satisfaction with the current treatment in relation to treatment target

Satisfaction with the effect of the current treatment was in 88 % of the visits in the BeSt-study (Fig. 1b) and 81 % in the IMPROVED-study (Fig. 2b). In the BeSt-study, if the DAS was low, rheumatologists were more often satisfied with the current treatment effect (<1.6: 76.48 (53.67–108.98) and ≥1.6–2.4: 10.07 (7.95–12.76)) (Table 4). In the IMPROVED-study, DAS <1.6 resulted in more satisfaction with the treatment effect (26.06 (20.68–32.84)). If the DAS increased, rheumatologists became less satisfied with the current treatment effect in both studies (BeSt-study: 0.09 (0.08–0.11) and IMPROVED-study 0.07 (0.06–0.08)).

Satisfaction with the next treatment step was 76–84 % during the first year of the BeSt-study (Fig. 1d). During 5 years, the satisfaction of rheumatologists with the next treatment step increased to 86 %. In the IMPROVED-study, this question was not asked to the rheumatologists after the second year. During the first year, 76–86 % of the rheumatologists were satisfied with the treatment step, and in the second year this percentage slightly decreased to 80 % (Fig. 2d).

## Discussion

Treatment-to-target is recommended for treatment of patients with RA, but in daily practice it may be challenged by rheumatologists’ willingness to conform to protocolled treatment adjustments aiming at a predefined target. Non-adherence may diminish the effect of a treat-to-target protocol, but both the protocol and the target may diminish adherence. In this study, we investigated the target effect. We compared adherence to two treatment protocols, one aimed at achieving low disease activity (DAS ≤2.4, in the BeSt-study) and one aiming at achieving DAS-remission (DAS <1.6, in the IMPROVED-study), and found that protocol adherence was higher in the DAS ≤2.4 targeted study. Protocol adherence decreased over time in both studies, but more in the DAS <1.6 targeted study. This was not particularly due to antagonism towards the required tapering of treatment as soon as DAS <1.6 was achieved at a four-monthly evaluation time point, as we found that protocol violations occurred more often against treatment intensification than against tapering. In the DAS ≤2.4-steered study, which had more delayed tapering strategies, this was equal. In both studies, violations were associated with rheumatologists’ disagreement with how the measured DAS represented actual disease activity, or with the next treatment step, and with a patient’s VASgh that was ≥20 mm higher than the physicians VAS-disease activity. In the DAS <1.6-steered study, apparent discrepancies between number of swollen and painful joints measured ESR, and reported VASgh were associated with more violations compared to the DAS ≤2.4-steered study.

Following a protocol that aims at a stricter treatment target is more difficult. It may be felt that there is no additional clinical benefit to be achieved, or there are perceived risks, for instance of side effects and/or higher costs, which may reduce physician’s compliance. In addition, there may be doubt whether the composite score used to measure disease activity does represent actual disease activity [15]. This is certainly suggested by our finding that rheumatologists reported more often that they felt the measured DAS overestimated actual disease activity in a DAS <1.6-steered treatment protocol compared to a DAS ≤2.4-steered treatment protocol. When in the DAS <1.6-steered study, the DAS approaches the target, rheumatologists also appear more sensitive to apparent discrepancies between subjective and (semi)objective representations of disease activity and reluctant to steer by DAS alone. Still, median differences between measured DAS and target DAS, relative to whether or not the rheumatologist adhered to the protocol, may represent a tendency of the rheumatologists to try to stay closer to the target DAS <1.6 than they did to the target DAS ≤2.4. This suggests a learning effect, where between the start of the BeSt-study in 2000 and the start of the IMPROVED study in 2007, rheumatologists have conformed and became accustomed to DAS targeted treatment and agree with the idea that DAS remission is a target worth aiming for. In addition, they also seem to agree that relatively rapid and complete drug tapering in patients with early RA or undifferentiated RA, should be tried as soon as DAS <1.6 is achieved, as protocol violations were less often against low DAS than against high DAS.

We are the first to compare treatment targets in DAS-steered treatment protocols in early arthritis patients by comparing protocol adherence and protocol violations in a long follow-up period of 5 years, having access to two such studies with similar technical protocols but aiming at different DAS targets, conducted by largely the same rheumatologist. Both studies were embedded in daily practice in the rheumatologists’ office, and our results may reflect their willingness to conform to targeted treatment protocols outside clinical trials. There were a lot of differences between the two studies that make it difficult to compare them head-to-head. The IMPROVED-study also included UA patients next to RA patients whereas in the BeSt study all patients had RA. In the BeSt-study, patients had a more severe disease and the target was not strict compared to the IMPROVED-study. Furthermore, RA was associated with more low DAS-protocol violations. This may indicate that RA is considered as a more severe disease than UA.

Our results suggest that a DAS-steered treatment can be implemented in daily practice. If there is a defined target, the chance to achieve the target is eventually high. However, a stricter treatment target is more difficult to implement in daily practice, because rheumatologists will be content with a slightly higher DAS if they feel it does not represent actual disease activity. Perceived risks of the required steps may reduce physicians’ adherence. This however can negatively influence patient outcomes.

The COBRA study aimed at DAS-remission, and showed comparable protocol violations during 6 months follow-up (24 %) [16]. Recently, a sub-analysis of the NEO-RACo study showed that physicians’ better adherence to a protocol steered at modified ACR remission [17] was associated with better clinical outcomes and a lower rate of prescription of biologic DMARD in later years [18]. Also in other diseases, physicians’ adherence to a treatment protocol was associated with better outcomes [19–22]. It is clear that a stricter DAS target may not be achievable in all patients. Patient factors, type of disease, comorbidities, and drug-related risks may affect components of the DAS or prevent further treatment adjustments. Ideally, the optimal treatment target is clear for each patient, allowing individualized treatment. [23].

In conclusion, adherence to two DAS-steered treatment protocols was high, but adherence decreased over 5 years. This decrease was more distinct in a DAS <1.6 steered protocol, where violations were more likely if the physician disagreed with the measured DAS. Protocol violations were then more often against required treatment intensification than against required tapering, whereas with a target DAS ≤2.4 this was balanced. Also, in a DAS <1.6-steered protocol violations occurred more often in case of potential discrepancies between detected joint swelling, pain and ESR. Our results may indicate that adherence to DAS-steered protocols appears to depend at least in part on the height of the target, and in addition on how physicians perceive the DAS reflects RA activity. Targeted treatment is important to achieve the best possible outcomes for RA patients. It would be preferable to combine the trend to set ever stricter treatment targets with the benefits of an individualized approach.

## Electronic supplementary material

Figure 1 BeSt-study flow chart treatment strategies. *AZA* azathioprine 2-3 mg/kg/day; *CSA* ciclosporin A 2.5 mg/kg/day; *depomedrol* 3 injections of 120 mg in week 1, 4 and 8; *Gold* 50 mg/week; *HCQ* hydroxychloroquine 200 mg/day; *IFX* infliximab, dosages once per 8 weeks; leflunomide 20 mg/day; *MTX* methotrexate, dosage per week; *Pred* prednisone 7.5 mg/day unless indicated otherwise; *SSA* sulphasalazine 2000 mg/day. (GIF 168 kb)

High resolution image (EPS 1029 kb)

Figure 2 IMPROVED-study flow chart. *MTX* methotrexate, 25 mg/week; *HCQ* hydroxychloroquine; *SSZ* sulphasalazine. Colours: orange = prednisone, green = MTX, dark blue = treatment according to opinion rheumatologist (TAR), aqua = HCQ, yellow = SSZ, purple = adalimumab biweekly, double thickness purple = adalimumab weekly, grey = protocol not followed as required but remained in follow-up (outside of protocol, OOP). All patients started with MTX and prednisone, tapered from 60 to 7.5 mg/day in 7 weeks. After 4 months if patients were in remission (DAS <1.6) prednisone was tapered to MTX monotherapy. If patients were not in remission they were randomized to arm 1 (MTX 25 mg/week, HCQ 400 mg/day, SSZ 2000 mg/day and prednisone 7.5 mg/day) or arm 2 (MTX 25 mg/week plus adalimumab 40 mg/2 weeks). Every 4 months if patients were in remission, the medication was tapered or stopped and if patients were not in remission, the medication was intensified or restarted. (GIF 58 kb)

High resolution image (EPS 589 kb)
