# Supplementary figures and images for: Rheumatologists’ adherence to a disease activity score steered treatment protocol in early arthritis patients is less if the target is remission
Source: Clin Rheumatol. 2016 Sep 28;36(2):317–26. doi: 10.1007/s10067-016-3405-8 (PMC5290046; doi:10.1007/s10067-016-3405-8)

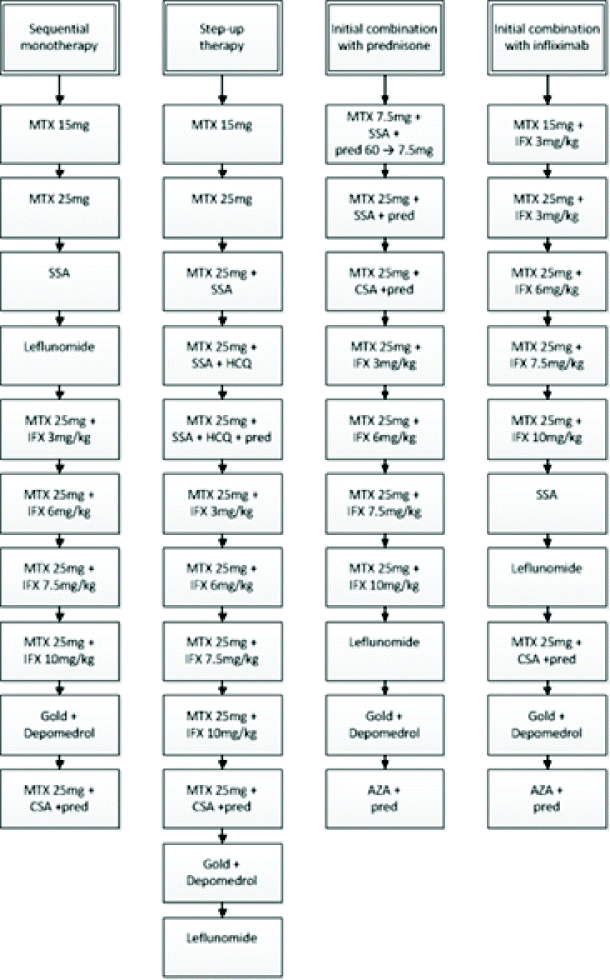

Supplement: Supplementary file 1 — BeSt-study flow chart treatment strategies. AZA azathioprine 2-3 mg/kg/day; CSA ciclosporin A 2.5 mg/kg/day; depomedrol 3 injections of 120 mg in week 1, 4 and 8; Gold 50 mg/week; HCQ hydroxychloroquine 200 mg/day; IFX infliximab, dosages once per 8 weeks; leflunomide 20 mg/day; MTX methotrexate, dosage per week; Pred prednisone 7.5 mg/day unless indicated otherwise; SSA sulphasalazine 2000 mg/day. (GIF 168 kb) [file 10067_2016_3405_Fig3_ESM.gif]

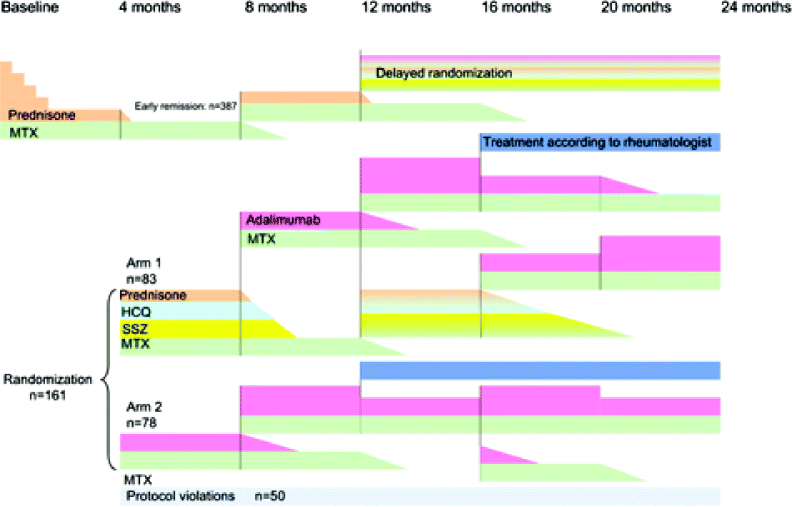

Supplement: Supplementary file 3 — IMPROVED-study flow chart. MTX methotrexate, 25 mg/week; HCQ hydroxychloroquine; SSZ sulphasalazine. Colours: orange = prednisone, green = MTX, dark blue = treatment according to opinion rheumatologist (TAR), aqua = HCQ, yellow = SSZ, purple = adalimumab biweekly, double thickness purple = adalimumab weekly, grey = protocol not followed as required but remained in follow-up (outside of protocol, OOP). All patients started with MTX and prednisone, tapered from 60 to 7.5 mg/day in 7 weeks. After 4 months if patients were in remission (DAS <1.6) prednisone was tapered to MTX monotherapy. If patients were not in remission they were randomized to arm 1 (MTX 25 mg/week, HCQ 400 mg/day, SSZ 2000 mg/day and prednisone 7.5 mg/day) or arm 2 (MTX 25 mg/week plus adalimumab 40 mg/2 weeks). Every 4 months if patients were in remission, the medication was tapered or stopped and if patients were not in remission, the medication was intensified or restarted. (GIF 58 kb) [file 10067_2016_3405_Fig4_ESM.gif]
